# *ACTB* Loss-of-Function Mutations Result in a Pleiotropic Developmental Disorder

**DOI:** 10.1016/j.ajhg.2017.11.006

**Published:** 2017-12-07

**Authors:** Sara Cuvertino, Helen M. Stuart, Kate E. Chandler, Neil A. Roberts, Ruth Armstrong, Laura Bernardini, Sanjeev Bhaskar, Bert Callewaert, Jill Clayton-Smith, Cristina Hernando Davalillo, Charu Deshpande, Koenraad Devriendt, Maria C. Digilio, Abhijit Dixit, Matthew Edwards, Jan M. Friedman, Antonio Gonzalez-Meneses, Shelagh Joss, Bronwyn Kerr, Anne Katrin Lampe, Sylvie Langlois, Rachel Lennon, Philippe Loget, David Y.T. Ma, Ruth McGowan, Maryse Des Medt, James O’Sullivan, Sylvie Odent, Michael J. Parker, Céline Pebrel-Richard, Florence Petit, Zornitza Stark, Sylvia Stockler-Ipsiroglu, Sigrid Tinschert, Pradeep Vasudevan, Olaya Villa, Susan M. White, Farah R. Zahir, The DDD Study, Adrian S. Woolf, Siddharth Banka

**Affiliations:** 1 Division of Evolution and Genomic Sciences, School of Biological Sciences, Faculty of Biology, Medicine, and Health, The University of Manchester, M13 9PL Manchester, UK; 2 Manchester Centre for Genomic Medicine, St. Mary’s Hospital, Manchester University Foundation NHS Trust, Health Innovation Manchester, M13 9WL Manchester, UK; 3 Division of Cell Matrix Biology and Regenerative Medicine, School of Biological Sciences, Faculty of Biology, Medicine and Health, The University of Manchester, M13 9PL, Manchester, UK; 4 East Anglian Medical Genetics Service, Department of Clinical Genetics, Addenbrooke’s Hospital, CB2 0QQ, Cambridge, UK; 5 Cytogenetics Unit, Casa Sollievo della Sofferenza Hospital, 71013 San Giovanni Rotondo, Italy; 6 Center for Medical Genetics, Ghent University Hospital, 9000 Ghent, Belgium; 7 Quantitative Genomic Medicine Laboratories (qGenomics), 08950 Barcelona, Spain; 8 Clinical Genetics Department, Guy’s Hospital, SE1 9RT London, UK; 9 Center for Human genetics, Katholieke Universiteit Leuven and University Hospital Leuven, B-3000 Leuven, Belgium; 10 Medical Genetics, Bambino Gesù Pediatric Hospital, IRCCS, 00165 Rome, Italy; 11 Department of Clinical Genetics, Nottingham City Hospital, NG5 1PB Nottingham, UK; 12 Department of Paediatrics, School of Medicine, University of Western Sydney, NSW 2751, New South Wales, Australia; 13 Department of Medical Genetics, University of British Columbia, BC V6T 1Z4 Vancouver, Canada; 14 Servicio de Pediatría, Hospital Viamed Santa Ángela de la Cruz, 41014 Sevilla, Spain; 15 West of Scotland Genetics Service, Queen Elizabeth University Hospital, G51 4TF Glasgow, UK; 16 South East of Scotland Clinical Genetic Department, Western General Hospital, EH4 2XU Edinburgh, UK; 17 Service d’Anatomie Pathologique, Hôpital Pontchaillou, University Rennes 1, 35000 Rennes, France; 18 Service de Génétique Clinique, Centre de Référence “Maladies Rares” CLAD-Ouest, Hôpital SUD, University Rennes 1, UMR 6290, 35000 Rennes, France; 19 Sheffield Clinical Genetics Service, Sheffield Children’s NHS Foundation Trust, Western Bank, S20 1NZ Sheffield, UK; 20 Service de Cytogénétique Médicale, Centre Hospitalier Régional Clermont-Ferrand, 63000 Clermont-Ferrand, France; 21 Service de Genetique Clinique, Centre Hospitalier Régional Lille, 59000 Lille, France; 22 Victorian Clinical Genetics Services, Murdoch Children’s Research Institute, VIC 3052 Melbourne, Australia; 23 Zentrum Medizinische Genetik, Medical University of Innsbruck, 6020 Innsbruck, Austria; 24 Department of Clinical Genetics, University Hospitals of Leicester NHS Trust, Leicester Royal Infirmary, LE1 5WW Leicester, UK; 25 Department of Paediatrics, University of Melbourne, VIC 3010, Melbourne, Australia; 26 Qatar Biomedical Research Institute, Hamad Bin Khalifa University, 34110 Doha, Qatar; 27 Wellcome Centre for Cell-Matrix Research, Division of Cell-Matrix Biology and Regenerative Medicine, School of Biological Sciences, Faculty of Biology, Medicine, and Health, University of Manchester, M13 9PL Manchester, UK; 28 Wellcome Trust Sanger Institute, CB10 1SA Cambridge, UK; 29 Department of Nephrology, Royal Manchester Children’s Hospital, Manchester Academic Health Science Centre, M13 9WL Manchester, UK

**Keywords:** ACTB, β-actin, malformations, developmental disorder, chromatin

## Abstract

*ACTB* encodes β-actin, an abundant cytoskeletal housekeeping protein. In humans, postulated gain-of-function missense mutations cause Baraitser-Winter syndrome (BRWS), characterized by intellectual disability, cortical malformations, coloboma, sensorineural deafness, and typical facial features. To date, the consequences of loss-of-function *ACTB* mutations have not been proven conclusively. We describe heterozygous *ACTB* deletions and nonsense and frameshift mutations in 33 individuals with developmental delay, apparent intellectual disability, increased frequency of internal organ malformations (including those of the heart and the renal tract), growth retardation, and a recognizable facial gestalt (interrupted wavy eyebrows, dense eyelashes, wide nose, wide mouth, and a prominent chin) that is distinct from characteristics of individuals with BRWS. Strikingly, this spectrum overlaps with that of several chromatin-remodeling developmental disorders. In wild-type mouse embryos, β-actin expression was prominent in the kidney, heart, and brain. *ACTB* mRNA expression levels in lymphoblastic lines and fibroblasts derived from affected individuals were decreased in comparison to those in control cells. Fibroblasts derived from an affected individual and *ACTB* siRNA knockdown in wild-type fibroblasts showed altered cell shape and migration, consistent with known roles of cytoplasmic β-actin. We also demonstrate that *ACTB* haploinsufficiency leads to reduced cell proliferation, altered expression of cell-cycle genes, and decreased amounts of nuclear, but not cytoplasmic, β-actin. In conclusion, we show that heterozygous loss-of-function *ACTB* mutations cause a distinct pleiotropic malformation syndrome with intellectual disability. Our biological studies suggest that a critically reduced amount of this protein alters cell shape, migration, proliferation, and gene expression to the detriment of brain, heart, and kidney development.

## Main Text

Developmental disorders (DDs) are thought to affect 2%–5% of individuals and are genetically heterogeneous.^1^ They range from isolated internal organ malformations and intellectual disability to complex syndromic presentations. In developed economies, congenital malformations are one of the leading causes of death among children and account for almost 25% of neonatal deaths.^2^ DDs constitute a large proportion of the life-long global health burden in terms of medical expenditure, hospitalizations, and mortality.^2^ Accurate diagnosis and better mechanistic understanding are key to improving medical management.

**Figure 1:**
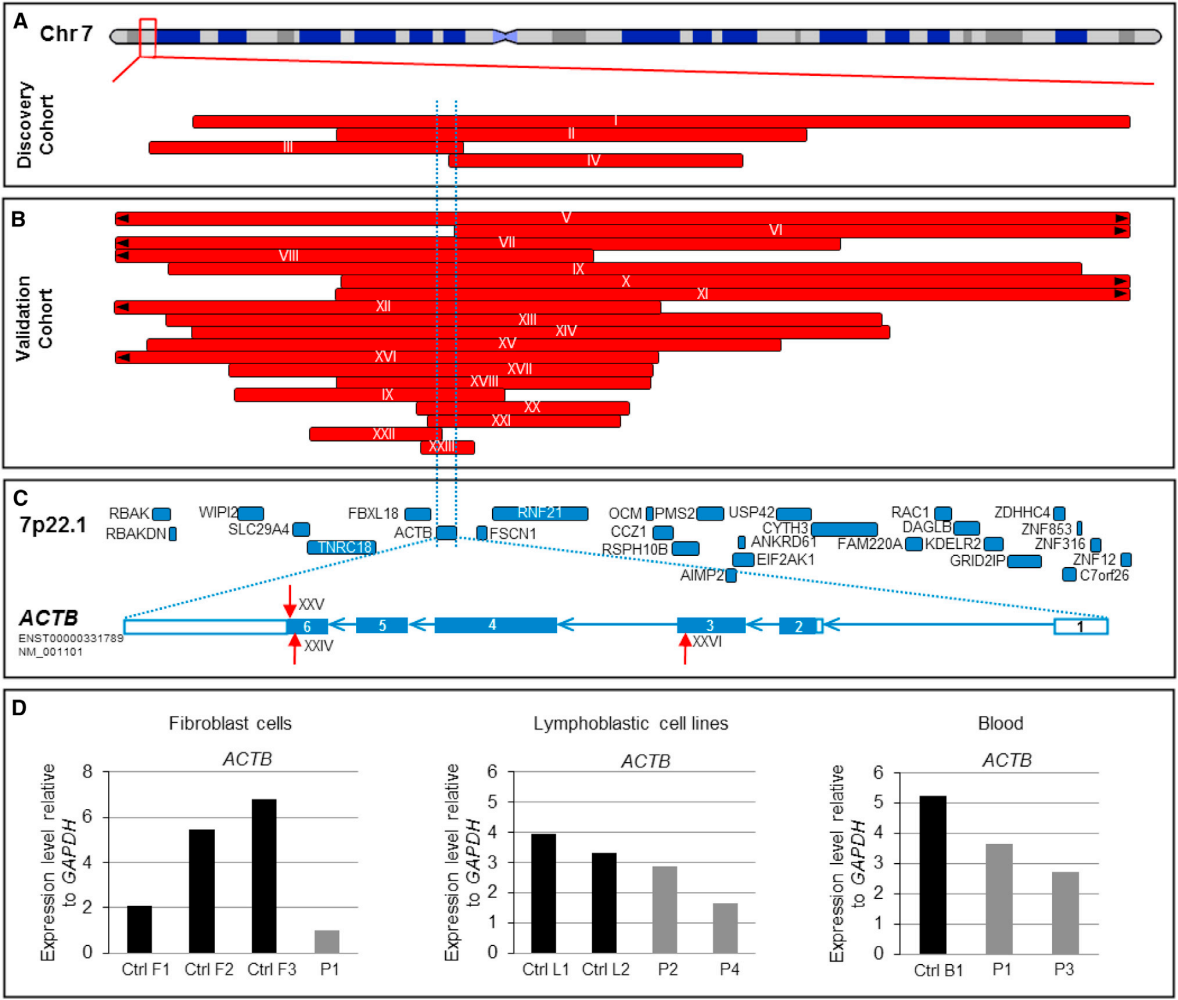
*ACTB* Loss-of-Function Mutations Result in Reduced Expression (A) Discovery cohort—a representation of chromosome 7 with four small 7p22.1 deletions identified in the discovery cohort. The red bars represent the extent of genomic deletions, and the associated family number is linked to Table 1. (B) Validation cohort—the 19 small 7p22.1 deletions identified in the validation cohort. The red bars represent the extent of genomic deletions, and the associated family number is linked to Table 1. (C) Intragenic *ACTB* mutations—the location of the known protein-coding genes on 7p22.1 are shown in blue boxes. *ACTB* is highlighted by two flanking dashed blue lines. The lower panel shows the exon structure of *ACTB.* The boxes represent the *ACTB* exons, and the blue arrows denote the introns. Note that ACTB is transcribed from the reverse strand. The filled (blue) and unfilled sections of the exons denote translated and untranslated regions of the gene, respectively. Location of mutations in the three affected individuals is shown with red arrows—one with a small frameshift deletion (XXIV: p.Ser368LeufsTer13), one with a nonsense mutation (XXV: p.Lys373Ter) and one with *de novo* frameshift mutation (XXVI: p.Leu110ArgfsTer10). The transcript ID is NM_001101.3. (D) *ACTB* loss-of-function mutations result in reduced gene expression—quantitative real-time polymerase chain reaction (qRT-PCR) analysis of *ACTB* transcript levels relative to *GAPDH* in fibroblasts, LCL and blood samples (sample P1 is from IVa, P2 is from XI, P3 is from II, and P4 is from XXII). Cells were collected by centrifugation, and total RNAs were extracted with the RNeasy Mini kit (QIAGEN) according to the manufacturer’s protocol. The qRT-PCR reactions were performed on a Bio-Rad CFX394 Real-Time system (Bio-Rad) with Power SYBR Green PCR Master mix (Applied Biosystems). The expression of each target gene was evaluated via a relative quantification approach (-ΔCT method), and *GAPDH* was used as the internal reference for human genes.

**Figure 2:**
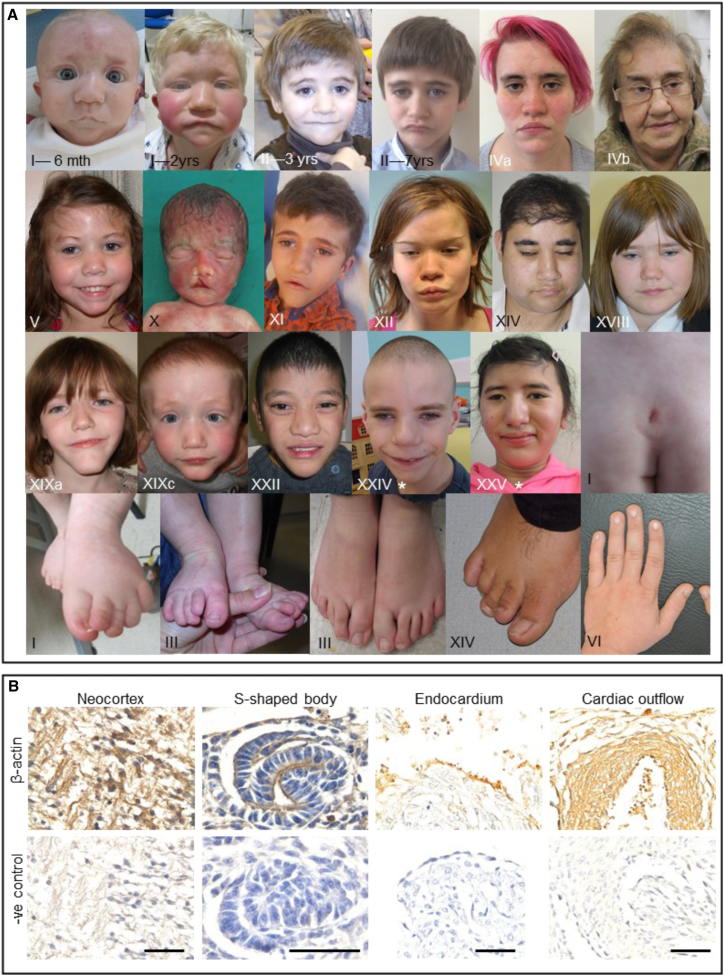
*ACTB* Loss-of-Function Mutations Result in a Recognizable Phenotype (A) Facial gestalt and physical anomalies with *ACTB* loss-of-function mutations—the facial features of a number of individuals who had 7p22.1 deletions or point mutations (marked with ^∗^) in *ACTB* were remarkably similar to each other; they had wavy interrupted eyebrows, dense eyelashes, a wide nose, a wide mouth, and a prominent chin. Several affected individuals in this cohort demonstrated overlapping toes, small nails, and spinal anomalies such as sacral dimples. (B) Immunohistochemistry of embryonic day 14 mice. The top row shows β-actin immunostaining (brown) in neurons in the brain (neocortex), epithelia of kidney tubules (S-shaped body), and the heart (endocardium and cardiac outflow). The bottom row shows sections with primary antibody omitted so that the specificity of the above signals is shown. Whole embryos at day 14 (E14) were fixed in 4% paraformaldehyde and embedded in paraffin. Sections of 5 μm were cut and mounted on polylysine-coated glass slides. Endogenous peroxidase was quenched by incubation with hydrogen peroxide (0.3% solution in PBS). Embryos were heated at 95°C for 5 minutes in sodium citrate (pH 6) for antigen exposure. Rabbit anti-β-actin (1:250, Abcam ab8227) was applied to tissue sections over night at 4°C. Goat anti-rabbit (1:200) was applied for 1 hr at room temperature and revealed with the ABC Elite kit (Vector) followed by DAB staining (Vector) and hematoxylin counter-stain (scale bar: 50 μm).

Rare copy-number variations associated with human DDs can provide insights into single-gene conditions and their molecular mechanisms.^3^, ^4^, ^5^, ^6^, ^7^ From more than 15,000 individuals who underwent clinical array comparative genomic hybridization for suspected genetic DDs at our center, we identified five individuals from four families with 7p22.1 deletions (Figure 1A; Table S1) and an overlapping phenotype (families I–IV in Table 1; Figure 2A). *ACTB* [MIM: 102630] was the only gene common to all four deletions, leading us to hypothesize that *ACTB* haploinsufficiency leads to a distinct clinical syndrome.

We ascertained 26 additional individuals from 19 families with likely or definitely pathogenic 7p22.1 deletions that were <3Mb and encompassed *ACTB* (Figure 1B; Table S1). Next, we interrogated data from 4,293 trios in the Deciphering Developmental Disorders study^1^ for *de novo* nonsense or frameshift variants in all known protein-coding genes on chromosome 7p22.1. We identified two *ACTB* point mutations, c.1097dupG; p.Ser368LeufsTer13 and c.1117A>T; p.Lys373Ter, in two children (NM_001101.3; ENST00000331789) (Figures 1C; Table S1). Finally, we identified another individual with a c.329delT; p.Leu110ArgfsTer10 *ACTB* point mutation in the CAUSES Study by using the analytical pipeline described previously.^8^ The procedures followed were in accordance with the ethical standards of the responsible committee on human experimentation (institutional and national), and proper informed consent was obtained.

Multiple lines of evidence establish *ACTB* loss-of-function mutations as a cause of a pleiotropic clinical syndrome. First, of the genes on human chromosomal region 7p22.1, *ACTB* is the only one with a high-probability loss-of-function intolerance (pLI) score^9^ and low residual variation intolerance score (RVIS)^10^ as well as a low haploinsufficiency index (HI)^11^ (Table S2). Second, it was the only gene deleted within the minimum critical region in both the discovery and validation cohorts of individuals with 7p22.1 deletions. Importantly, we also identified three individual with *ACTB* point mutations that, like the deletions, are expected to produce a heterozygous null *ACTB* genotype. Third, mutations, including all point mutations, were proven to have arisen *de novo* in 12 individuals. Biological parentage was proven in the three individuals who were identified via exome sequencing. In all multiplex families, the deletions segregated with the phenotype. Fourth, the striking phenotypic convergence in a large cohort ascertained on the basis of genotyping followed by reverse phenotyping^12^ rules out possibility of a chance association. Collectively, in this cohort of 33 individuals from 25 unrelated families we observed a high frequency of developmental delay, apparent intellectual disability, internal organ malformations (affecting heart, kidneys, spine, and palate, among others), growth retardation, and facial dysmorphism (interrupted eyebrows, dense eyelashes, wide nose, wide mouth, and a prominent chin) (Figure 2A; Table 1).

**Table 1:** Clinical Features of Individuals with Deletions, Stop-Gained, or Frameshift Mutations Involving *ACTB*

**Case ID**	**Inheritance**	**Gender**	**Age (Years)**	**Prenatal and Neonatal History**	**PN Growth Retardation**	**Microcephaly**	**Motor Delay**	**Speech Delay**	**DD/ ID**	**Behavioral, Psychiatric, and Neurological Features**	**Malformations and Physical Anomalies**	**Additional Comments**
**Discovery Cohort**												

I	DN	M	4	SGA and feeding difficulties	Y	N	Y	Y	mod	possible absence and focal seizures	VSD with tortuous aortic arch, horseshoe kidney, cryptorchidism, BL inguinal hernia, deep sacral dimple and BL single palmar creases	early-onset hypothyroidism, limitation of joint mobility and cutis marmorata
II	DN	M	7	SGA and feeding difficulties	Y	N	Y	Y	mild	sociable, empathetic, hand flapping tendency and attention deficit	Rt pelvic kidney, Rt inguinal hernia and scoliosis.	GOR, asthma and allergies.
III	DN	M	7	SGA, polycythaemia, jaundice and hypoglycaemia. Congenital CMV infection	Y	Y	N	Y	Mild	attention deficit, echolalia and tantrums.	inguinal hernia, cryptorchidism, proximally placed second toes and microcornea.	perineal and scalp abscesses, recurrent chest and ear infections, allergies and nephrotic syndrome.
IVa	Mat	F	32	SGA	Y	N	Y	Y	mod	empathetic personality.	scoliosis	glaucoma, asthma, and eczema
IVb	U	F	68	U	U	U	U	U	mild	U	horseshoe kidney with multiple cysts	hiatus hernia

**Validation Cohort**												

V	DN	F	13	SGA and feeding difficulties	Y	N	Y	Y	mod	sociable personality, mild ventriculomegaly, and multifocal small T2 hyperintensitites in the cerebral white matter	VSD, PDA, BL 5th finger clinodactyly, BL 2-3 & Lt 3-4 toe syndactyly	N
VI	U	M	20	N	Y	N	N	Y	mild	stress intolerance.	short and broad uvula, broad halluces, short distal phalanx of finger and toes, small nails, and 5th finger clinodactyly.	frequent otisis media, GH deficiency and limitation of joint mobility
VII	DN	F	12	SGA, hypotonia and feeding difficulties	Y	Y	Y	Y	mod	emotional problems and hypotonia	tricuspid valve dysplasia, 2-3-4 fingers and 2-3 toes syndactyly	BL severe SNHL and dorsal hypertrichosis
VIII	DN	M	7	SGA, hypotonia and feeding difficulties	Y	N	Y	Y	sev	Thin CC, septum pellucidum cyst, megacisterna magna, mild ventricular dilation and subependymal heterotopia	BL CLAP, VSD, Lt extra nipple, hypospadias, UL cryptorchidism and sacral dimple	cutis marmorata; additional *de novo* 1.65 Mb loss 7:6243891-7889083
IX	DN	M	6	hypotonia and feeding difficulties	Y	Y	Y	Y	mod	cortical and subcortical atrophy	atrial septal defect and BL inguinal hernia	GOR
X	DN	F	0 (fetus)	antenatal ultrasound: cleft lip and palate, septum pellucidum agenesis	NA	NA	NA	NA	NA	absent septum pellucidum and hydrocephalus	horseshoe kidney and non-midline CLAP	N
XI	DN	M	6	hypotonia and feeding difficulties	N	N	Y	Y	mod	sociable personality, ASD, hypotonia, possible seizures and periventricular heterotopias	BL absent thumbs, bowed radii, shortened forearms, chordee and parameatal cyst	GOR and hypermetropia. Published Shimojima et al., 2016^14^ ( #3)
XII	U	F	21	N	N	N	Y	Y	Mild	sociable personality, attention deficit, trichotillomania and seizures in early childhood	VSD, scoliosis, clinodactyly, short thumbs, camptodactyly fetal finger pads, tapering finger and 2-3 toe syndactyly	asthma, eczema, reduced elbow and shoulder extension, BL keratoconus and early Lt posterior subcapsular cataract
XIII	DN	F	2	SGA	Y	Y	Y	Y	mild	N	supra-valvular aortic stenosis	N
XIV	DN	M	20	hypotonia and feeding difficulties	U	U	Y	Y	mod	empathetic personality and ASD.	Pierre Robin sequence, Dislocated radial heads, Rt single palm crease, fetal finger pads, narrow fingernails, brachydactyly and toe camptodactyly	marked external rotation of hips, HL. Lt Astigmatism. Thin scalp hair. Published in Shimojima et al., 2016^14^ (#2)
XV	DN	M	4 months	SGA, polyhydramnion	Y	Y	NA	NA	NA	hypertonia	micropenis, retrognathia, dysplastic ears	additional paternally inherited 370 Kb loss 14:27438600-27806980
XVI	U	M	38	SGA	Y	N	N	Y	mild	ASD, drooling and seizures.	thoracic kyphosis and BL inguinal hernia	delayed puberty and recurrent sinusitis
XVIIa	mat	F	2	hypotonia and feeding difficulties.	N	N	Y	Y	mild	hypotonia	N	chronic respiratory infections
XVIIb	U	F	34	N	Y	Y	N	Y	mild	acute psychosis age 10, attention deficit and infarction right frontal cortex	fifth-finger clinodactyly.	chronic sinusitis
XVIII	DN	F	13	SGA	N	N	Y	Y	mild	anxious personality and pathological demand avoidance	gap between palate and posterior wall of pharynx.	recurrent urinary tract infections
XIXa	mat	F	7	N	N	Y	N	N	mod	N	TAPVD, scoliosis, tapering digits and fetal finger pads.	BL HL
XIXb	mat	F	5	SGA	N	Y	Y	Y	mild	N	atrial septal defect, sacro-coccygeal sinus and tethering of spinal cord	BL HL
XIXc	mat	M	2	N	N	N	Y	Y	mod	N	VSD, horseshoe kidney, VUR and hydronephrosis	BL HL and Rt eye strabismus
XIXd	U	F	37	N	N	N	N	N	mild	N	N	N
XXa twin I	mat	F	4	born at 26 week gestation, oligohydramnios and dizygotic twins; SGA, hypotonia and feeding difficulties	Y	Y	Y	Y	mod	attention deficit, CC hypoplasia and cortical atrophy	N	both have BL moderate SNHL and strabismus; additional paternally inherited 1.78Mb dup 16:88335976-90111263; Published in Shimojima et al., 2016^14^ (#4 & 5)
XXb twin II	mat	F	4		Y	Y	Y	Y	mod	attention deficit	N	
XXc	U	F	27	SGA	Y	Y	Y	Y	mild	Attention deficit	N	published in Shimojima et al., 2016^14^ (mother of #4 & 5)
XXI	pat	M	4	feeding difficulties	N	Y	Y	Y	mod	ASD and hypotonia	N	hypermobile joints; additional paternally inherited 16p11.2dup.
XXII	U	M	11	hypotonia and feeding difficulties	Y	Y	Y	Y	mod	affectionate personality, self-harming tendency, ASD, hypotonia and gray matter heterotopia	renal cortical cysts, congenital diaphragmatic hernia, scoliosis, fetal finger pads and brachydactyly	severe GOR, eczema and BL mixed HL.
XXIII	U	M	23	feeding difficulties and hypoglycemia	N	N	N	Y	mod	anxious personality and schizophrenia.	unilateral renal agenesis, ectopic testis, rectal stenosis, umbilical hernia, high palate, kyphosis and scoliosis	N

**Point Mutations**												

XXIV	DN	M	12	feeding difficulties	Y	Y	Y	N	mod	hyperactivity and dystonia	tracheesophageal fistula, esophageal atresia, overlapping toes, short foot and tapered fingers and pectus excavatum.	hypertrichosis
XXV	DN	F	14	feeding difficulties	N	Y	N	Y	mild	N	lytic lesions in both parietal bones suggestive of congenital parietal foramina	extra-skin folds on abdomen and back
XXVI	DN	M	18	N	N	N	Y	Y	mild	slight social inhibition	atrial septal defect and pectus deformity	BL SNHL, distal interphalangeal joint contracture of left 5^th^ finger, BL knee flexion contractures, mild weakness in ankle plantar flexion; dihydropyrimidine dehydrogenase deficiency due to compound heterozygous pathogenic mutations in *DYPD*; additional maternally inherited duplications (chr10:117216305-117530316 and chr10:117616145-118447670)

This table summarizes the clinical features of individuals in the discovery or validation cohort and point mutations involving *ACTB* (ASD, autistic spectrum disorder; BL, bilateral; CC, corpus callosum; CLAP, cleft lip and palate; DD, developmental delay; DN, *de novo*; HL, hearing loss; F, female; GH, growth hormone; GOR, gastresophageal reflux; ID, intellectual disability; Lt, left; M, male; mat, maternal/mother; mod, moderate; N, no or none known; NA, not applicable; PDA, patent ductus arteriosus; PN, postnatal; pat, paternal/father; Rt, right; sev, severe; SGA, small for gestational age; SNHL, sensorineural hearing loss; TAPVD, total anomalous pulmonary venous drainage; U, unknown; VSD, ventricular septal defect; VUR, vesicoureteric reflux; and Y, yes).

We performed qRT-PCR and measured *ACTB* mRNA expression in fibroblasts, lymphoblastic cell lines (LCLs), and fresh blood. Expression levels in affected individual-derived cells were consistently lower than in control samples (Figure 1D). Similar findings have been reported in *Actb*^+/−^ mice^13^ and in immortalized lymphocytes derived from one individual with a 7p22.1 microdeletion.^14^

Next, we analyzed β-actin expression in embryonic day 14 mice, a stage when organogenesis is ongoing; this stage is anatomically similar to the late first trimester of human gestation. β-actin was prominent in organs affected by the syndrome: cortical neurons and choroid plexus epithelia in the brain; differentiating tubules of the metanephric kidney; and the epicardium, endocardium, and muscle in the outflow tract of the heart (Figures 2B). Notably, it was not expressed uniformly in all embryonic cell types, even from within an organ.

**Figure 3:**
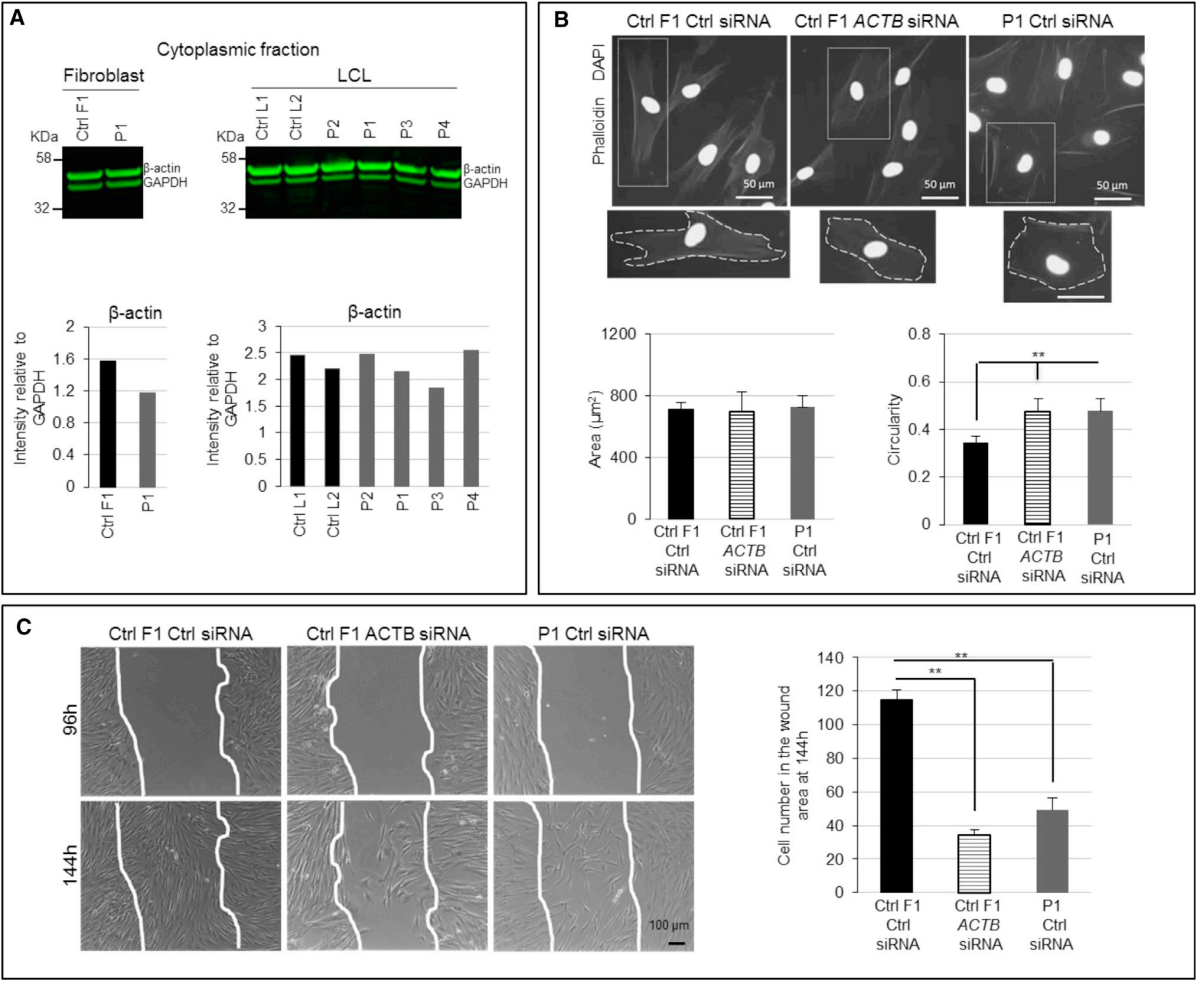
*ACTB* Loss-of-Function Mutations Induce Abnormalities of Cellular Morphology and Reduced Migration (A) Immunoblots of cytoplasmic β-actin. No consistent differences were detected in the cytoplasmic β-actin amounts in the fibroblasts and LCLs of affected individuals versus controls (sample P1 is from IVa, P2 is from XI, P3 is from II, and P4 is from XXII). Immunoblotting for β-actin was performed on the cytoplasmic protein fraction, and GAPDH was used as a loading control. Protein samples were isolated using NE-PER nuclear and cytoplasmic extraction reagents (ThermoScientific). 8–10 mg of protein extracts were loaded into the polyacrylamide gel Bolt 10% Bis-Tris Plus Gels (Invitrogen). The membranes were incubated with specific anti-beta actin (ab8227, Abcam) and anti-GAPDH (5174S, Cell Signaling) overnight at 4°C. After washes, the membranes were incubated with a secondary fluorescently labeled goat anti-rabbit antibody (IRDye 800CW Li-Cor), and signal was developed with an Odyssey CLX imaging machine. (B) Fibroblast morphology. *ACTB*-deficient cells were found to be significantly more circular than non-deficient cells. Phalloidin and DAPI immunostaining was performed in wild-type fibroblasts transfected with 30 nM control siRNA (ON-TARGETplus non-targeting pool, Fisher) and *ACTB* siRNA (SMARTpool ON TARGET plus ACTB siRNA, Dharmacon) and affected-individual fibroblasts transfected with control siRNA. Cells were fixed with 4% paraformaldehyde for 15 minutes at room temperature. After blocking solution was washed out, antibody Texas Red-X Phalloidin (T7471, Life Technologies) was applied for 1 hr at room temperature in the dark. Samples were stained with DAPI (4083S, Cell Signaling Technology) for 5 min. Representative pictures show marked difference in the morphology of β-actin-deficient cells (scale bar: 50 μm). Enlargement of cells is shown in the lower panels. Dashed lines show the outline of the cell boundary. The left bar chart shows that there was no significant difference in the area of each of the cell groups (in μm^2^) as calculated with ImageJ software. The right bar chart shows the increased circularity of the *ACTB*-deficient fibroblasts as calculated with ImageJ software (n = 4; ^∗∗^p < 0.01). Values of 1 and 0 stand for a perfect circle and a line, respectively. Error bars indicate mean ± 1 SD. (C) Fibroblast migration. *ACTB*-deficient cells had impaired migration. A migration assay was performed in wild-type fibroblasts transfected with control siRNA and *ACTB* siRNA and affected-individual fibroblasts transfected with control siRNA. 96 hr after transfection, a wound was generated in the confluent monolayer of fibroblasts via a p200 pipet tip. Cells were washed with phosphate-buffered saline so that any debris created by the wound would be removed. The first image of the wound was taken with a phase-contrast microscope, and marking the plate under the capture image field created a reference point. Cells were incubated at 37°C in a humidified 5% CO_2_ incubator for 2 days. After the incubation time, a second image was taken. For quantifying the migration of cells, the cells that crossed into the wound area were counted. Representative pictures show reduced migration in β-actin-deficient cells (scale bar: 100 μm). The bar chart shows that the numbers of cells in the central wound area are significantly lower in the β-actin-deficient cells. Data are shown as the mean of absolute cell numbers at 144 hours from two wells of two independent experiments (n = 4; ^∗^p < 0.05, ^∗∗^p < 0.01). Error bars indicate mean ± 1 SD.

β-actin is essential for a number of cytoplasmic functions, such as regulation of cell shape and migration.^13^, ^15^, ^16^ We performed an immunoblot and quantified the amount of β-actin in the cytoplasmic protein fraction of affected individual cells, but no consistent differences in β-actin expression were observed in affected individual-derived cells versus controls (Figure 3A). However, affected individual-derived fibroblasts were significantly more circular than controls, although there was no alteration in the average cell size (Figure S1). Their migration was also severely impaired in comparison to that of control fibroblasts (Figure 3C). Importantly, silencing-RNA (siRNA)-mediated downregulation of *ACTB* in control fibroblasts (Figure S2) induced a similar increase in circularity (Figure 3B) and migration defects (Figure 3C), proving that the changes in cells with a 7p22.1 deletion were primarily due to *ACTB* haploinsufficiency.

**Figure 4:**
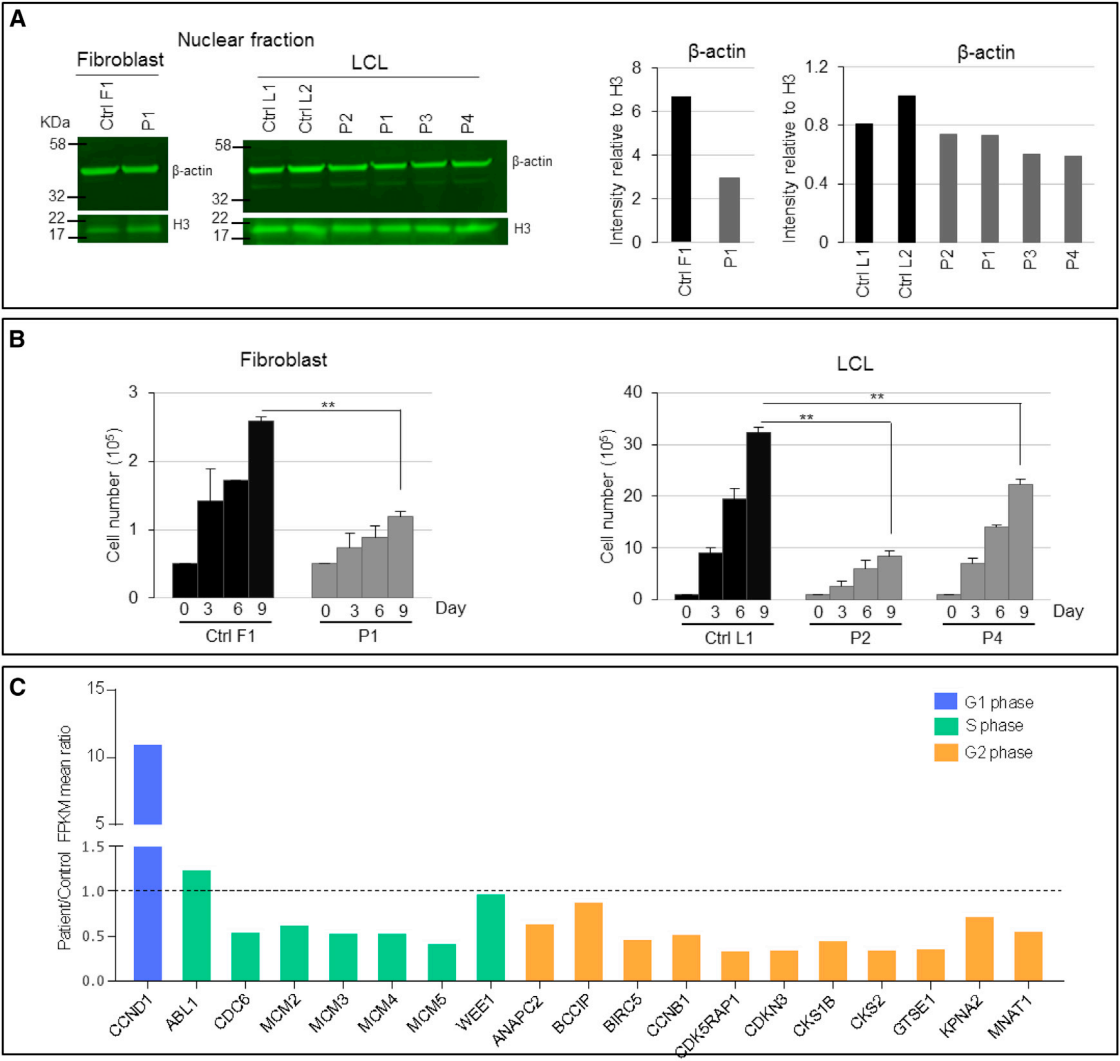
*ACTB* Loss-of-Function Mutations Lead to Reduced Expression of β-actin in the Nucleus, Reduced Cell Proliferation, and Dysregulation of Cell-Cycle Genes (A) Immunoblots of nuclear β-actin. Nuclear β-actin amounts in fibroblasts and LCLs from affected individuals were consistently lower than those of their respective controls (sample P1 is from IVa, P2 is from XI, P3 is from II, and P4 is from XXII). Immunoblotting for β-actin (ab8227, Abcam) was performed on the nuclear protein fraction with histone 3 (ab1791, Abcam) as a loading control. Visual inspection of the membranes and their intensity quantification revealed a consistent trend of decreased amounts of nuclear β-actin in fibroblasts and LCLs from affected individuals versus controls (sample P1 is from IVa, P2 is from XI, P3 is from II, and P4 is from XXII). (B) Fibroblast and LCL proliferation. Proliferation in cells derived from affected individuals was found to be significantly reduced. Quantification of the number of fibroblasts and LCLs was performed in a 12-well plate. Cells were plated in the presence of their growth medium and counted with a hemocytometer every 3 days for 9 days. Fibroblasts and LCLs from affected individuals proliferated significantly slowly in comparison with the control cells. Data are shown as the mean of absolute cell number from three wells of three independent experiments (n = 3; ^∗^p < 0.05, ^∗∗^p < 0.01). Error bars indicate mean ± 1 SD. (C) Expression of cell-cycle genes in LCLs. Cell-cycle genes were dysregulated in LCLs from affected individuals. The abundance of selected mRNAs in LCLs derived from individuals XI (P2) and XXII (P4) is shown relative to mean amounts in LCLs in two control individuals; measurements are in FPKM (fragments per kilo base of transcript per million mapped reads). Libraries were prepared for sequencing with the NEBNext Ultra Directional RNA Library Prep Kit for Illumina (New England Biolabs) according to the manufacturer’s instructions. Sequencing of a single 75 bp read was carried out on a NextSeq 500 sequencer (Illumina) according to the manufacturer’s protocols. An average of 34.9 million reads was generated per sample. For each sample, the RNA-Seq reads were aligned to the human reference GRCh37 with Tophat v2.1.0. Cufflinks v2.2.2 was used for assembling the aligned reads against UCSC hg19_refgene transcripts and for generating the relative expression levels, measured as FPKM, for each transcript within each sample. The expression of *CCND1* is more than 11-fold higher in cells of affected individuals than in controls. The expression of a majority of genes expressed in S and G2 phase is reduced in cells of affected individuals.

In the nucleus β-actin regulates gene expression, cell division, and proliferation.^17^, ^18^ Interestingly, in contrast to the cytoplasmic fraction, the nuclear protein fraction showed a reduced amount of β-actin in cells derived from affected individuals (Figure 4A). Interference with nuclear β-actin function directly correlates with levels of transcription and cell proliferation.^19^ Accordingly, we found significantly decreased cellular proliferation in affected individual-derived cells (Figures 4B and 4C). We examined expression of cell-cycle genes by using RNA-Seq on samples derived from two controls and two LCLs from affected individuals (Table S3). In cells from affected individuals, we detected 11-fold increased expression of *CCND1* [MIM: 168461], a key gene that encodes cyclin D1, which should be downregulated for cells to transition from G1 into the S phase (Figure 4C).^20^ This is consistent with the previous observation that β-actin inhibition leads to arrest in the G1 phase.^21^ Furthermore, the expression levels of several S- and G2-phase genes were lower in LCLs from affected individuals than in control LCLs (Figure 4C).

Although chromosome 7p22.1 deletions have been described in a small number of affected individuals previously, *ACTB* has not been conclusively proven to be the underlying gene responsible for the phenotype.^15^ We have now described numerous affected families, suggesting that the syndrome caused by loss-of-function *ACTB* mutations might have been under-recognized. Variation in the phenotype could be due to differences in the sizes of deletion causing loss of additional genes or long-range regulatory dysfunction or due to genetic background or environmental effects. Notably, two point mutations in exon 6 are predicted to escape nonsense-mediated decay, and one in exon 3 is predicted to cause protein truncation. Still, it is remarkable that the phenotype of these individuals is similar to that of individuals with *ACTB* deletions as opposed to BRWS [MIM: 243310],^22^ which suggests that the dosage of full-length β-actin plays an important role in normal human development.

All individuals in our cohort displayed developmental delay and apparent intellectual disability. In several individuals, expressive speech was severely affected. A trusting, empathetic, or sociable personality was reported independently in many individuals. Some were reported to have autism-spectrum or attention-deficit hyperactivity disorders. Actin filaments are a major structural component of synapses and are critical for synaptic plasticity, which directly influences neurodevelopment, cognitive performance, and social behavior.^23^, ^24^ This could explain the developmental and behavioral phenotype of affected individuals.

Among ten individuals who were investigated with brain magnetic-resonance imaging, eight (80%) were detected to have some abnormality, including gray-matter heterotopias in two individuals (Table 1). This is consistent with neuronal migration defects, prominent expression of β-actin in cortical neurons of developing mice, and our findings of defects in cellular migration in cells from affected individuals. Other neuroradiological features included cortical atrophy, cerebral white-matter hyperintensities, a thin corpus callosum, a septum pellucidum cyst, megacisterna magna, ventricular enlargement, and hydrocephalus. Seizures were reported in a minor subset of individuals, and one individual had dystonia. Delayed-onset generalized dystonia has been reported in monozygotic twins with an *ACTB* p.Arg183Trp mutation.^25^

Congenital cardiac anomalies such as ventricular and atrial septal defects, sub-aortic stenosis, tortuous arch, total anomalous pulmonary venous drainage, patent ductus arteriosus, tricuspid valve dysplasia, and ventricular dilatation were observed in 11 out of 22 (50%) individuals who were investigated by echocardiogram or post-mortem examination (Table 1). Major renal anomalies were detected in 7 out of 19 (36.8%) individuals whose renal ultrasound examination or post-mortem reports were available. These included four individuals with horseshoe kidneys, and one each with unilateral renal agenesis, pelvic kidney, and kidney cysts (Table 1). Vesicouretric reflux and hydronephrosis were also seen in some individuals. Those males, who lacked overt renal malformations, still had a high frequency of anomalies such as inguinal hernias, hypospadias, micropenis, urethral cyst and cryptorchidism. Spinal and palatal anomalies were also detected in six and four individuals, respectively. Collectively, our data show that internal organ malformations, particularly in the heart and kidneys, are much more frequent in individuals with loss-of-function *ACTB* mutations than in the general population.^26^, ^27^, ^28^, ^29^ A role of β-actin in morphogenesis is supported by our detection of this protein in the developing heart and kidney epithelia in mice. Furthermore, in cells from affected individuals, we showed altered cellular morphology, migration, and proliferation, which are all key developmental processes.

Although growth parameters were not available for all affected individuals, we noted growth retardation in 13 individuals. This could be related to β-actin’s role in cell growth and proliferation, which we also observed in the affected individual’s cells.^17^

The individuals described here have overlapping dysmorphism with interrupted wavy eyebrows, dense eyelashes, hypertelorism, a wide nose, a wide mouth, and a prominent chin. This is distinct from the typical facial dysmorphism of BRWS, although we did notice some overlapping features, such as a wide mouth and hypertelorism, in both groups. None of our individuals were detected to have agyria/pachygyria or coloboma, which are frequent in BRWS.^30^, ^31^ This further emphasizes that *ACTB* loss-of-function mutations cause a specific syndrome distinct from BRWS.

The facial characteristics of individual described here resemble those in several chromatin remodeling disorders, such as the interrupted eyebrows in Kabuki syndrome [MIM: 147920 and 300867]^32^, ^33^ and the typical lip configuration and large mouth seen in KBG syndrome [MIM: 148050]. β-actin is a part of chromatin remodeling complexes such as SWR1 and SWI-SNF,^18^ and mutations in its components cause Floating-Harbour syndrome [MIM: 136140]^34^ and Coffin-Siris syndrome [MIM: 135900],^35^ respectively. Of note, we detected decreased amounts of nuclear β-actin, along with dysregulated gene expression, in cells of affected individuals. The role of nuclear β-actin has been the subject of recent intense research,^36^, ^37^ and our results highlight the importance of its correct dosage in human development.

In summary, we have described, in 33 individuals, a pleiotropic disorder caused by haploinsufficiency of *ACTB*, which encodes for the most abundant eukaryotic cytoplasmic protein, β-actin. Although we have shown some of the consequences of reduced dosage of *ACTB in vitro*, further studies will be required to if we are to understand the mechanisms behind the phenotypes resulting from loss-of-function *ACTB* mutations. Notably, the mechanisms linking *ACTB* missense mutations with BRWS also remain unknown. The partial overlap of phenotypes of individuals with BRWS and *ACTB* loss-of-function mutations suggest that perhaps the underlying mechanism of BRWS is not just, as postulated, gain of function, but might also include some effects resulting from loss-of-function or dominant-negative effects. Researchers are increasingly recognizing that different mutations in the same gene can have different underlying genetic effects and thus can result in different phenotypes.^38^ Interestingly, missense *ACTG1* [MIM: 102560] (encoding the only other ubiquitously expressed actin, γ-actin) mutations also result in BRWS.^22^ However, we did not observe any distinct phenotype that could be attributed to *ACTG1* deletions (data not presented). Of note, the pLI score for *ACTG1* is 0.22, which decreases the likelihood that heterozygous loss-of-function mutations in this gene are a cause of an early-onset human disorder. Hence, these observations support overlapping but distinct cellular roles for the two ubiquitously expressed β- and γ-actins. Overall, our linked biology studies suggest that a critically reduced amount of β-actin alters cell shape, migration, proliferation, and gene expression to the detriment of brain, heart, and kidney development.

## Web Resources

ExAC Browser, http://exac.broadinstitute.org/gene/ENSG00000075624 and http://exac.broadinstitute.org/gene/ENSG00000184009
GenBank, http://www.ncbi.nlm.nih.gov/genbank/
OMIM, http://www.omim.org

## Supplemental Data

Document S1. Figures S1–S3 and Tables S1–S3

Document S2. Article plus Supplemental Data
