# Supplementary material for: ACTB Loss-of-Function Mutations Result in a Pleiotropic Developmental Disorder
Source: Am J Hum Genet. 2017 Dec 7;101(6):1021–33. doi: 10.1016/j.ajhg.2017.11.006 (PMC5812896; doi:10.1016/j.ajhg.2017.11.006)
Supplement: Document S1. Figures S1–S3 and Tables S1–S3 [file mmc1.pdf]

The American Journal of Human Genetics, Volume *101*

Supplemental Data

ACTB Loss-of-Function Mutations

Result in a Pleiotropic Developmental Disorder

Sara Cuvertino, Helen M. Stuart, Kate E. Chandler, Neil A. Roberts, Ruth Armstrong, Laura Bernardini, Sanjeev Bhaskar, Bert Callewaert, Jill Clayton-Smith, Cristina Hernando Davalillo, Charu Deshpande, Koenraad Devriendt, Maria C. Digilio, Abhijit Dixit, Matthew Edwards, Jan M. Friedman, Antonio Gonzalez-Meneses, Shelagh Joss, Bronwyn Kerr, Anne Katrin Lampe, Sylvie Langlois, Rachel Lennon, Philippe Loget, David Y.T. Ma, Ruth McGowan, Maryse Des Medt, James O'Sullivan, Sylvie Odent, Michael J. Parker, Céline Pebrel-Richard, Florence Petit, Zornitza Stark, Sylvia Stockler-Ipsiroglu, Sigrid Tinschert, Pradeep Vasudevan, Olaya Villa, Susan M. White, Farah R. Zahir, The DDD Study, Adrian S. Woolf, and Siddharth Banka

SUPPLEMENTARY MATERIAL

SUPPLEMENTARY FIGURES

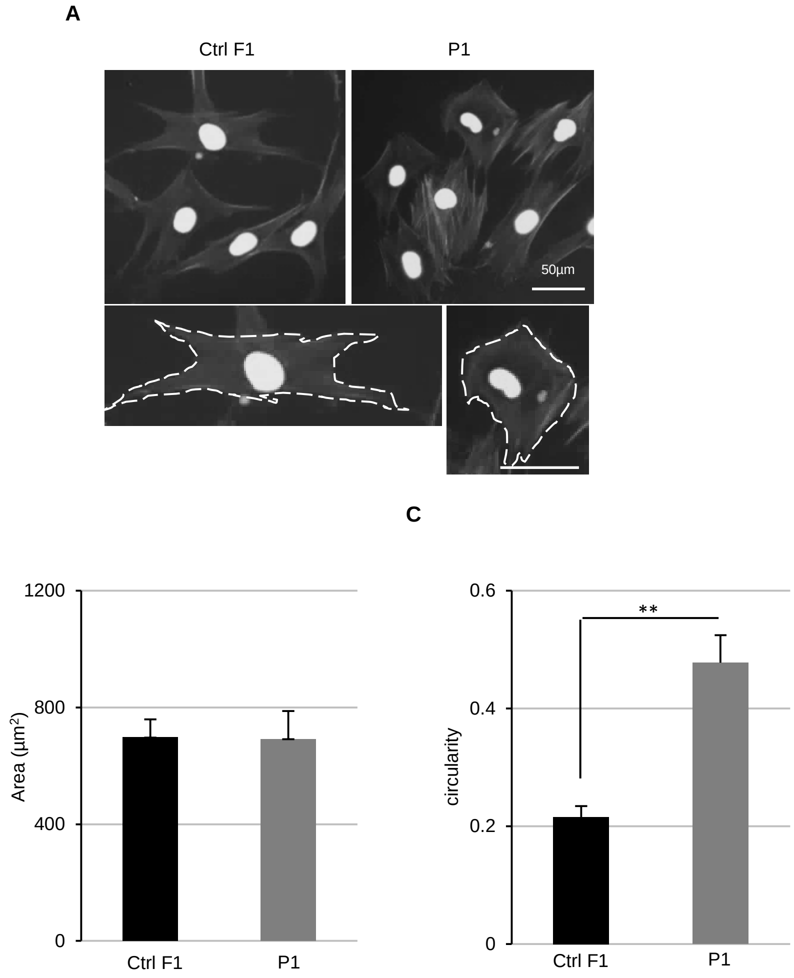

Figure S1: Analysis of fibroblast morphology without being treated with siRNA constructs. (A) Immunostaining of fibroblast cells stained for Phalloidin expression and DAPI (Scale bar: 50 µm; Ctrl F1: control fibroblast, P1: sample from IVa in Table 1). **(B)** Bar chart shows the cell area (µm^2) of control or affected individual fibroblast cells transfected with ACTB siRNA or control siRNA. Measurement has been done using ImageJ software. **(C)** Bar chart shows the circularity of control or affected individual fibroblast cells transfected with ACTB siRNA or control siRNA. Measurement has been done using ImageJ software (n=4; ** P<0.01). Circularity with a value of 1 indicates a perfect circle. As the value approaches 0, it indicates an increasingly elongated shape.

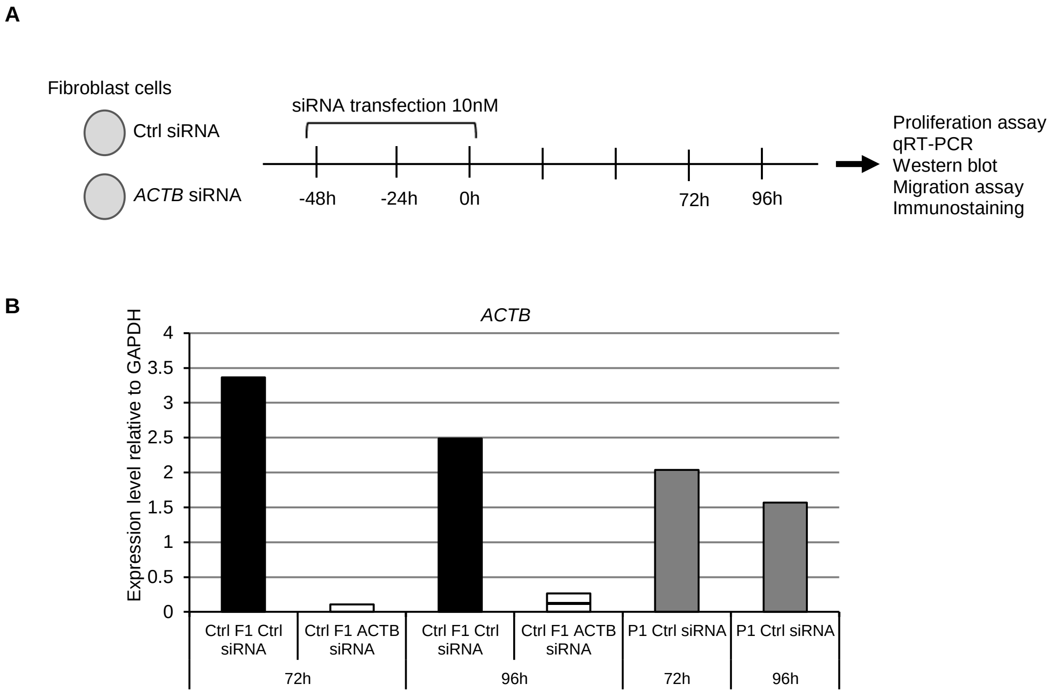

Figure S2: Cell transfection protocol and confirmation of *ACTB* knockdown. (A) Scheme of the protocol used to down-regulate *ACTB* with siRNA. Cells were transduced with 10nM siRNA constructs at -48h, -24h and 0h. Seventy-two and ninety-six hours later, cells were analysed by qRT-PCR, western blot and other down-stream assays. **(B)** Quantitative RT-PCR analysis of *ACTB* transcript level relative to *GAPDH* in fibroblasts samples transfected with siRNA against *ACTB* or control (Ctrl F1: control fibroblast, P1: sample from IVa).

SUPPLEMENTARY TABLES

Family	Origin	Decipher ID	Deletion coordinates (Hg19)	Size (Mb)
**Discovery Cohort**				
I	UK	271006	7:5151574-6745570	1.59
II	UK	256348	7: 5370613-6296827	0.93
III	UK	258437	7: 5057992- 5617869	0.56
IV	UK	255897	7: 5587489-6080286	0.49
**Validation Cohort**				
V	Canada	998	7: 3531608-7167693	3.64
VI	Austria	284613	7: 5592436-8416721	2.82
VII	Italy	254233	7: 3861164-6213355	2.35
VIII	Italy	256546	7: 3861164-5864755	2.00
IX	Spain	-	7: 5105131-6714753	1.61
X	France	268591	7: 5399371-6871084	1.47
XI	Belgum	253773	7: 5370746-6781213	1.41
XII	UK	252339	7: 4778156-5945860	1.17
XIII	Australia	-	7: 5184455-6348344	1.16
XIV	Australia	274371	7: 5151474-6296948	1.15
XV	Belgium	289362	7: 5048743-6146825	1.10
XVI	UK	272915	7: 4861557-5923456	1.06
XVII	Belgium	288265	7: 5233021-5926631	0.69
XVIII	UK	301216	7: 5370613-5925070	0.55
XIX	UK	259191	7: 5241760-5675100	0.43
XX	France	264408	7: 5492967-5864851	0.37
XXI	UK	283142	7: 5548368-5857259	0.31
XXII	Australia	-	7: 5359680-5568627	0.21
XXIII	France	-	7: 5536848-5617875	0.08
**Intragenic variants**				
XXIV	UK	263884	7: 5567409-5567409; c.1097dupG; p.(Ser368LeufsTer13)	
XXV	UK	267004	7: 5567390; c.1117A>T; p.(Lys373Ter)	
XXVI	Canada	-	NM_001101:exon3: c.329delT; p.(Leu110ArgfsTer10)	

Table S1: Genotypes of individuals described in this study.

Gene	Probability of Loss-of-function intolerance (pLI)	Residual Variation Intolerance score p0.1% (percentile)	haploinsufficiency index (HI index %)
ACTB	**0.94**	**-0.67 (16)**	**1.03**
AIMP2	0.00	-0.36 (29)	41.23
ANKRD61	NA	NA	85.91
C7orf26	0.83	-0.34 (31)	56.53
CCZ1	**0.99**	-0.16 (42)	39.44
CYTH3	0.36	**-0.54 (20)**	50.20
DAGLB	0.00	0.03 (56)	71.98
EIF2AK1	0.00	0.36 (75)	66.82
FAM220A	0.00	0.89 (89)	96.17
FBXL18	0.00	**-0.80 (12)**	62.82
FSCN1	0.78	-0.04 (50)	21.98
GRID2IP	0.31	NA	61.05
KDELR2	0.27	-0.38 (27)	26.59
OCM	0.00	0.44 (78)	46.73
PMS2	0.00	1.48 (95)	59.43
RAC1	0.57	-0.16 (41)	**0.72**
RBAK	0.01	0 (54)	79.68
RNF216	0.41	**-1.19 (6)**	36.49
RSPH10B	NA	NA	86.61
SLC29A4	0.00	**-0.99 (9)**	66.44
TNRC18	**1.00**	NA	61.51
USP42	**1.00**	0.52 (80)	78.60
IPI2	0.09	0.13 (63)	47.26
ZDHHC4	0.00	0.78 (87)	79.09
ZNF12	0.55	-0.2 (39)	73.57
ZNF316	NA	NA	NA
ZNF853	0.00	NA	84.37

Table S2: Scores for intolerance of loss of function and haploinsufficiency for protein coding genes located on human chromosome 7p22.1.

We compiled the LoF intolerance (pLI) score[1] from http://exac.broadinstitute.org/; Residual Variation Intolerance score (RVIS)[2] from http://genic-intolerance.org/ and haploinsufficiency index (HI)[3] from DECIPHER. All resources were accessed on 03/04/2017 (NA, Data not available; pLi>0.9; RVIS percentile of <20; and HI of <20 are highlighted in bold).

Gene name	Aggregate FPKM values						
	**P2**	**P4**	**$Mean_P$**	**C1**	**C2**	**$Mean_C$**	**$Mean_P$/ $Mean_C$**
**G1-phase**							
CCND1	3.9	8.0	6.0	0.5	0.6	0.5	11.1
**S-phase**							
ABL1	18.6	24.7	21.7	19.7	14.7	17.2	1.3
CDC6	4.1	15.0	9.5	23.0	10.8	16.9	0.6
MCM2	42.8	71.1	57.0	110.2	68.1	89.1	0.6
MCM3	32.8	57.1	45.0	87.5	77.7	82.6	0.5
MCM4	16.6	51.0	33.8	81.4	42.3	61.9	0.5
MCM5	57.6	76.5	67.1	166.0	142.6	154.3	0.4
WEE1	11.0	21.3	16.2	18.9	13.8	16.4	1.0
**G2-phase**							
ANAPC2	14.2	19.9	17.0	30	21.7	25.9	0.7
BCCIP	52.2	64.8	58.5	73.4	57.1	65.2	0.9
BIRC5	16.5	31.3	23.9	56.7	44.1	50.4	0.5
CCNB1	22.3	52.5	37.4	81.0	59.0	70.0	0.5
CDK5RAP1	14.0	12.5	13.3	17.6	57.6	37.6	0.4
CDKN3	23.7	23.7	23.7	59.7	70.7	65.2	0.4
CKS1B	8.6	8.6	8.6	16.4	19.9	18.1	0.5
CKS2	47.4	80.1	63.8	162.0	195.3	178.7	0.4
GTSE1	7.4	15.1	11.3	26.8	33.2	30.0	0.4
KPNA2	59.5	124.5	92.0	145.4	103.3	124.4	0.7
MNAT1	35.0	54.6	44.8	77.8	78.7	78.2	0.6

Table S3: Comparison of aggregate FPKM values in control and affected individual LCLs for genes expressed in G1, S and G2 phases of the cell cycle.

P2 is from XI, P4 is from XXII in Table 1 (FPKM, Fragments Per Kilobase of transcript per Million mapped reads).

SUPPLEMENTARY REFERENCES

1. Lek, M., Karczewski, K.J., Minikel, E.V., Samocha, K.E., Banks, E., Fennell, T., O'Donnell-Luria, A.H., Ware, J.S., Hill, A.J., Cummings, B.B. et al. (2016). Analysis of protein-coding genetic variation in 60,706 humans. Nature. 536, 285–91.

2. Petrovski, S., Wang, Q., Heinzen, E.L., Allen, A.S., Goldstein, D.B. (2013). Genic intolerance to functional variation and the interpretation of personal genomes. PLOS Genet. 9, e1003709.

3. Huang, N., Lee, I., Marcotte, E.M., Hurles, M.E. (2010). Characterising and predicting haploinsufficiency in the human genome. PLoS Genet. 6, e1001154.
